# Covid-19 mobility restrictions: impacts on urban air quality and health

**DOI:** 10.5334/bc.124

**Published:** 2021-09-02

**Authors:** Nahid Mohajeri, Alina Walch, Agust Gudmundsson, Clare Heaviside, Sadaf Askari, Paul Wilkinson, Michael Davies

**Affiliations:** UCL Institute for Environmental Design and Engineering, Faculty of the Built Environment, University College London, London, UK; Solar Energy and Building Physics Laboratory, École Polytechnique Fédérale de Lausanne, Lausanne, Switzerland; Department of Earth Sciences, Royal Holloway College, University of London, Egham, UK; UCL Institute for Environmental Design and Engineering, Faculty of the Built Environment, University College London, London, UK; Smart Cities Lab, Hybream Ltd, London, UK; Centre on Climate Change and Planetary Health & Department of Public Health, Environments and Society, London School of Hygiene and Tropical Medicine, London, UK; UCL Institute for Environmental Design and Engineering, Faculty of the Built Environment, University College London, London, UK

**Keywords:** air pollution, air quality, cities, Covid-19, environmental health, lockdown, machine learning, mobility, NO_2_, PM_2.5_, public health, transport, vehicles

## Abstract

In 2020, Covid-19-related mobility restrictions resulted in the most extensive human-made air-quality changes ever recorded. The changes in mobility are quantified in terms of outdoor air pollution (concentrations of PM_2.5_ and NO_2_) and the associated health impacts in four UK cities (Greater London, Cardiff, Edinburgh and Belfast). After applying a weather-corrected machine learning (ML) technique, all four cities show NO_2_ and PM_2.5_ concentration anomalies in 2020 when compared with the ML-predicted values for that year. The NO_2_ anomalies are –21% for Greater London, –19% for Cardiff, –27% for Belfast and –41% for Edinburgh. The PM_2.5_ anomalies are 7% for Greater London, –1% for Cardiff, –15% for Edinburgh, –14% for Belfast. All the negative anomalies, which indicate air pollution at a lower level than expected from the weather conditions, are attributable to the mobility restrictions imposed by the Covid-19 lockdowns. Spearman rank-order correlations show a significant correlation between the lowering of NO_2_ levels and reduction in public transport (*p* < 0.05) and driving (*p* < 0.05), which is associated with a decline in NO_2_-attributable mortality. These positive effects of the mobility restrictions on public health can be used to evaluate policies for improved outdoor air quality.

**Policy Relevance:**

Finding the means to curb air pollution is very important for public health. Empirical evidence at a city scale reveals significant correlations between the reduction in vehicular transport and in ambient NO_2_ concentrations. The results provide justification for city-level initiatives to reduce vehicular traffic. Well-designed and effective policy interventions (*e.g*. the promotion of walking and cycling, remote working, local availability of services) can substantially reduce long-term air pollution and have positive health impacts.

## 1 Introduction

The Covid-19-related nationwide restriction to human mobility, including two major lockdowns, in the UK in 2020 makes it possible, for the first time, to assess the effects of such great changes in mobility on air quality (Higham *et al*. 2020; Monks & Williams 2020; Vito *et al*. 2020; Thomas *et al*. 2021). Various measures to control air pollution emissions over recent decades have improved the general air quality, and associated health impacts in the UK (Carnell *et al*. 2019), so that in much of the UK the air quality meets the minimum European Union standards that have been in place since 2008 (Saunders *et al*. 2012). However, these standards are not satisfied for all pollutants. For example, only about one-quarter of the 43 zones into which the UK is divided for the purpose of air quality assessment meets the minimum standards for nitrogen dioxide (NO_2_) (DEFRA 2020).

Poor air quality has negative effects on human health, including worsening respiratory and cardiovascular problems many of which are fatal (Zivin & Neidell 2018; Quarmby *et al*. 2019; WHO 2013a, 2013b). It is estimated that millions of people die every year from illnesses that are largely due to air pollution. For example, air pollution was the fourth leading risk factor for mortality worldwide in 2019, with ambient air pollution contributing to 6.67 million deaths globally (Health Effects Institute 2020). The European Environmental Agency (EEA) estimates that in 2014 some 78,000 premature deaths in 41 European countries could be attributed to excessive exposure to NO_2_, and as many as 428,000 to exposure to particulate matter PM_2.5_ (EEA 2020). Air pollution of various types also contributes to greenhouse effects and thus to global warming (Seinfeld & Pandis 2016; Smedley 2019). Harmful air pollutants include particulate matter (PM_2.5_ and PM_10_), NO_2_, nitric oxide (NO), carbon monoxide (CO), ozone (O_3_), lead (Pb) and benzene (C_6_H_6_). These pollutants are normally most common in urban areas, particularly in those with heavy traffic or industry, and also contribute to indoor air pollution (Ferguson *et al*. 2021). In the UK, the pollutants that are of most concern to health for the general population are PM_2.5_, NO_2_ and O_3_.

In view of the high number of attributed premature deaths and numerous non-fatal health issues related to these pollutants, it is of great importance to assess to what extent changes in human mobility (*e.g*. the use of cars, public transport and active travel) could reduce their concentrations in the air. The pollutants have many sources, but among the important ones are generation through human mobility, particularly fossil fuel-powered vehicles. The well-monitored Covid-19-related restrictions in human mobility in 2020 (particularly for driving and public transport) provide important information on the impact of mobility changes on the concentrations of these air pollutants.

During the Covid-19 pandemic, there have been extensive human mobility restrictions in many countries, including the UK. This paper focuses on changes in the concentration of two main air pollutants during the Covid-19-related human mobility restrictions in the UK for the entire year 2020, including the two lockdowns in 2020. The selected pollutants are NO_2_ and PM_2.5_. Both have adverse effects on human health, are strongly related to transport emissions (particularly NO_2_) and may be regarded as representative of a larger group of pollutants. In the UK, exposure to PM_2.5_ is estimated to lead to 29,000 excess deaths per year (COMEAP 2010); when including mortality from NO_2_, this figure is 34,000 deaths per year (RCP 2016).

The principal aim of this study is to quantify the change in ambient pollutant concentrations (NO_2_ and PM_2.5_) during the Covid-19 human mobility restrictions (using a weather-corrected machine learning (ML) technique), with a focus on the above four cities as case studies. For the detailed analysis, four major cities, namely Greater London, Cardiff, Edinburgh and Belfast, were selected. The analysis should provide a better understanding of how changes in human mobility-related activities in each city affect air quality. The year 2020 includes two major lockdowns (in spring and autumn). For comparison, this study includes the winter months January–February before any restrictions on human mobility were in place. Another aim is to explore the implications of the results for human health in urban areas, and to make approximate estimates of the fraction of annual all-cause mortality that can be associated with each pollutant for 2020, in comparison with preceding years.

## 2 Data Pre-Processing And Methods

### 2.1 Mobility Data

Data on human mobility in 2020 were compiled and analysed using publicly available smartphone data from Apple (*https://covid19.apple.com/mobility*). The mobility data are divided into the following categories based on means of transport: walking, driving (*e.g*. cars and lorries) and public transport (*e.g*. buses, passenger trains and underground). Apple’s mobility data are based on location data of Apple’s map services and can be used to help mitigate the spread of Covid-19 and provide information on the effects of the various restrictions and lockdowns on human mobility. The data derive from the number of requests by users for directions and are given for each day. All the data are shared on an aggregated level; there is no maintained and stored history of the mobility behaviour of individual users.

The mobility data through 2020 are compared with a baseline period, which is based on the requests by users for directions on 13 January 2020 and given in percentages. This was selected as a reference date by Apple partly because two weeks later, on 30 January, the World Health Organisation (WHO) classified Covid-19 as a ‘Public Health Emergency of International Concern’ (PHEIC). In the Apple data, days are defined as midnight to midnight using Pacific time.

**Figure 1:**
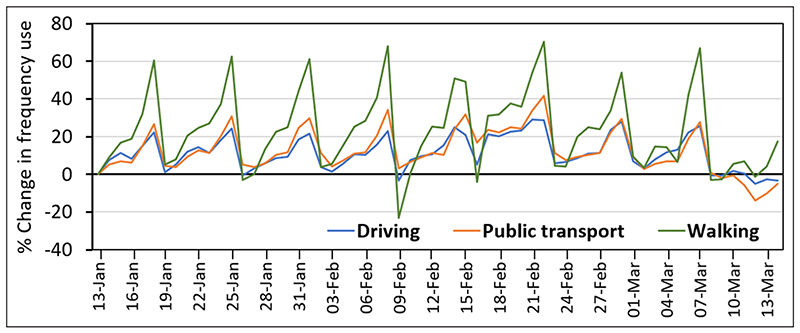
Bias in mobility data due to normalisation by a single day (provided by Apple), namely Monday, 13 January, the first day in the dataset. Source: https://covid19.apple.com/mobility/.

**Figure 2:**
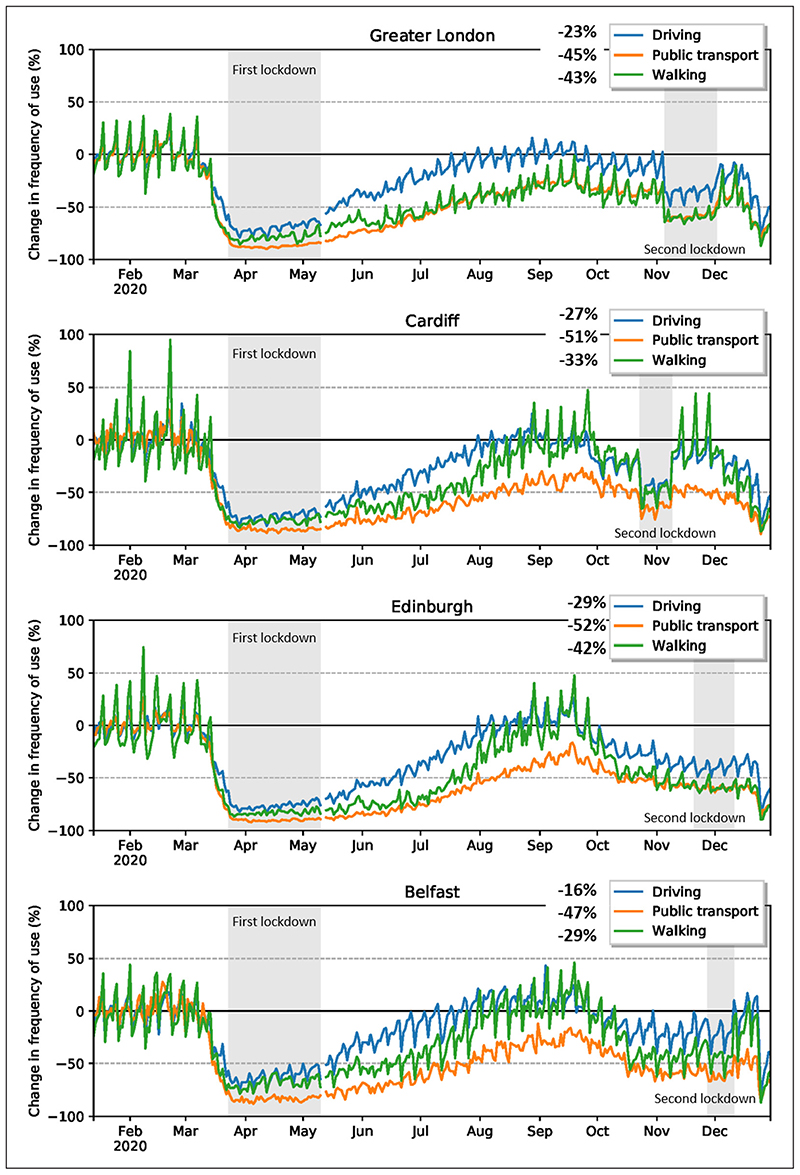
Changes in direction requests (driving, public transport, walking) from 13 January to the end of December 2020 (%). *Note:* The mobility changes for lockdown periods (first and second) are shown in dark grey. The Apple mobility data are normalised by taking 62 days from 13 January to 14 March as the baseline. Source: https://covid19.apple.com/mobility/.

As indicated, the mobility data as provided by Apple are normalised by a single day, namely Monday, 13 January, the first day in the dataset. However, nearly all the following days until mid-March have higher mobility data recorded. Thus, Monday, 13 January is statistically the day with the lowest mobility (Figure 1). This causes some bias in the mobility data. To remove this bias, the data are normalised over the entire period before the effects of Covid-19 restrictions are considered in the UK mobility data. This period is defined from Monday, 13 January to Sunday, 14 March. While the official lockdown in the UK only started on 23 March, a steep decrease in mobility is visible in the data from 14 March following lockdown measures in several European countries. To obtain mobility-reduction data with the pre-Covid mean at zero, the normalisation is performed as:

(1) $$m_{\text{norm},i,d}=\frac{m_{0,i,d}}{m_{pc,i}}–1$$

where *m_norm,i,d_* denotes the normalised mobility reduction for transport means *i* (driving, walking, public transport) on day *d*; *m_0,i,d_* is the original mobility data (with *m_0,i,0_* = 1 on 13 January); and *m_pc,i_* is the pre-Covid mean mobility, given by:

(2) $$m_{pc,i}=\frac{1}{N_{\rho C}}\sum_{d\in PC}m_{0,i,d}$$

where *PC* denotes the pre-Covid period from 13 January to 14 March, with *N_PC_* = 62 days. The mobility data obtained after applying the above pre-processing steps in Python is shown in Figure 2.

### 2.2 Air Pollutants

At some sites in the UK, the data collection goes back to 1972 with high-resolution hourly information. High levels of data availability mean that it is easy to compare the data from 2020 with earlier dates from the same sites, as is done here, where 2020 data are compared with those of each of 2010–19, inclusive. The Automatic Urban and Rural Network (AURN) measurements meet the standards set by the European Ambient Air Quality Directive 2008/50/EC (EU, 2008). AURN measures many pollutants including NO_2_ and PM_2.5_, which are the focus in the present study.

DEFRA (UK Air) classifies the monitoring sites into several different categories depending on their location. These categories are as follows: rural background, suburban background, suburban industrial, urban background, urban industrial and urban traffic. Urban traffic refers primarily to stations located close to roadsides. For the four cities, the number of stations used for the daily data is as follows: London, 16; Cardiff, two; Edinburgh, two; and Belfast, two. Of the London stations, 13 stations measure NO_2_ and 11 measure PM_2.5_. For the yearly averages as part of the health impact assessment, however, only stations in the category of urban background are used, as these are more representative of population exposure. In this category there are five stations for London, one for Cardiff, one for Edinburgh and one for Belfast.

### 2.3 Meteorological Data

The compiled daily meteorological data for the four cities are air temperature, precipitation and wind speed. These data were compiled for each day of 2020, as well as for the daily averages of the period 2010–19. The results are presented in Figures S1–S3 in Appendix A in the supplemental data online. The data used are from Meteostat, which provides access to meteorological data through a JavaScript Object Notation (JSON) Application Programming Interface (API) and a bulk data interface (*https://meteostat.net/en/station*). The database contains data from thousands of weather stations worldwide, which regularly report observations and statistics to Meteostat. Meteostat uses its own climate model to project observations and statistics of single weather stations on any geographical point. Therefore, matching weather stations around a reference point are weighted based on their three-dimensional distance and adjusted to the respective altitude.

### 2.4 Methods

#### 2.4.1 Time-series analysis

While there were suggestions to the public to reduce unnecessary travel earlier in 2020, the first formal lockdown due to Covid-19 in the UK was on 23 March 2020 (the ‘stay at home’ policy). This lockdown was nationwide and occurred at the same date and was of equal duration in all four cities, namely from 23 March to 10 May (when the ‘stay at home’ policy changed to the ‘stay alert’ policy). By contrast, the second lockdown was not at the same time in all the cities, as a tier system was introduced. In Greater London it was from 5 November to 2 December, in Cardiff from 23 October to 9 November, in Edinburgh from 20 November to 11 December, and in Belfast from 27 November to 11 December. Thus, not only were the second lockdowns at different times but also of different durations in these cities.

To analyse the temporal variations of air pollutants during the mobility restrictions in the four cities, daily mean concentrations of NO_2_ and PM_2.5_ were obtained for each day of the year in 2020 as well as for each day of the period 2010–19. To estimate the relative change (given as percentage), the two following items were calculated: (1) the difference between daily average pollutant levels for January–December 2020 and the average of those for a 10-year baseline (2010–19); and (2) the difference between daily average pollutant levels for the first lockdown (23 March–10 May; the same for all cities) and the second lockdown (varies between cities) for 2020 and the average of those for the 10-year baseline (2010–19). Data pre-processing for both daily mean concentrations data and daily meteorological data was made using Python.

#### 2.4.2 Machine learning (ML) weather correction

To consider the effects of meteorological variability on the measured air pollution, two ML algorithms are used—linear regression (LR) and gradient boosting regression (GBR)—to predict what air pollutant concentrations would be expected in the absence of any mobility restrictions in 2020. GBR, a popular ML algorithm, can be used for both regression and classification problems. The objective of any supervised learning algorithm such as GBR is to define a loss function and minimise it (Natekin & Knoll 2013). The mean squared error (MSE) is an example of loss function. Whereas random forests (RFs) build an ensemble of deep independent trees (using bagging technique), GBR builds an ensemble of shallow trees in a sequence, with each tree-learning improving the previous one (using a boosting technique). Both ML algorithms were trained on five-year historical data (daily air pollutant concentrations and daily metrological data) for the cities (consistent datasets for all cities is available only for the past five years). For Cardiff, however, temperature was the only meteorological data available. The following features in the training process are considered: (1) yearly and daily values for air pollutant concentrations (for 2015–19) to account for weekly cycles as well as for long-term trends; (2) temperature, precipitation and wind speed (for 2015–19); and (3) temperature, precipitation and wind speed offset by three days. The offset meteorological features allow for some time lags between weather conditions and pollutant concentrations to be modelled. All variables show significant correlations with pollutant concentrations. The predicted air pollutant concentrations are in μg m^−3^ and relative change in percentages. The feature importance for the four cities and for NO_2_ and PM_2.5_ are shown in Appendix B in the supplemental data online (Figure S4).

#### 2.4.3 Spearman rank-order correlation

To obtain the correlations between changes in mobility (vehicle driving and public transport use) and predicted air pollutant levels, the procedure was as follows. First, the daily air pollutant concentrations (NO_2_ and PM_2.5_) were aggregated to weekly means so as to smooth the data. The smoothing makes the long-term trends in the datasets clearer. Second, a Spearman rank-order correlation was used to assess the monotonic relationships (linear and non-linear) (*e.g*. Upton & Cook 1996; Corder & Foreman 2014). The statistical distributions of the changes in concentrations of pollutants and the changes in human mobility are not assumed to be normally distributed. Thus, parametric tests—where the parameters refer to the population of normal distributions—are not suitable, hence the use of non-parametric tests (distribution-free tests) such as Spearman’s (cf. Shaw & Wheeler 1985; Upton & Cook 1996).

## 3 Results

### 3.1 Changes in Mobility

Driving, public transport and walking all show a sharp and broadly similar decrease during the first lockdown in the four cities. The maximum measured change (Figure 2; negative values indicate a decrease) was in public transport, –92% (Edinburgh), followed closely by Greater London (–90%) and Cardiff (–89%), whereas the maximum changes in walking was –87% and in driving was –83% (Edinburgh). The changes are much less noticeable in the second lockdown and occur at different times in different cities, for the reasons given above. A summary of the mobility changes for each of the four cities during 2020, with a focus on the percentage decreases during the first and second lockdowns, is given in Figure 2 (maximums) and Table 1 (averages).

#### 3.1.1 Greater London

During the first lockdown there was a sharp decrease in driving, use of public transport and walking (Figure 2). The maximum measured change in public transport was –90%, in walking was –85% and in driving was –80%. This decrease occurred following the formal lockdown on 23 March and the mobility stayed at a much reduced level throughout the lockdown period, from 23 April to 10 May. Following gradual relaxation after 10 May of the restrictions to human mobility, the rise in driving was the fastest (Figure 2). Driving reached its pre-lockdown values—the baseline—before the second lockdown, while walking and public transport remained well below the baseline until the end of the year. The second lockdown in Greater London was from 5 November to 2 December (Figure 2 and Table 1). During this lockdown the maximum measured change in public transport was –63%, in walking was –66% and in driving was –49%. All these modes of transport stayed low during the lockdown, and then gradually rose in December until the third lockdown (at the end of December/beginning of January 2021). (Note that the third lockdown is not considered in this paper.)

For Greater London the average change in the first lockdown period was –86% for public transport, –78% for walking and –69% for driving (Table 1). For the duration of the second lockdown, the corresponding changes were –58% for public transport, –59% for walking and –36% for driving. For comparison, for the entire year, since 13 January 2020, the average changes were as follows: –45% for public transport, –43% for walking and –23% for driving. Thus, in all cases for Greater London the restrictions to human mobility had, for these three parameters, the greatest effect on the use of public transport, and least effect on driving.

#### 3.1.2 Cardiff

The first lockdown Cardiff showed an even greater decrease in driving than in Greater London (Figure 2). The maximum measured change in public transport was –89%, in walking was –83% and in driving was –82%. Figure 2 shows that these reductions in public transport, walking and driving were largely maintained through the lockdown period. After 10 May, both driving and walking rose in harmony to their pre-lockdown (baseline) values, whereas public transport rose much less and remained far below the pre-lockdown values until the end of the year. In this respect, the development of the mobility modes following the first lockdown is very different from that of Greater London, but similar to that observed for Edinburgh and Belfast (Figure 2). This difference between the cities as regards the use of public transport is presumably largely due to the necessity of such use in Greater London in contrast with the much smaller cities (and thus with shorter, and commonly walkable, distances) of Cardiff, Edinburgh and Belfast.

During the second lockdown in Cardiff, from 23 October to 9 November (Figure 2 and Table 1), the maximum measured change in public transport was –76%, in walking was –65% and in driving was –60%. All these modes of transport stayed low during the lockdown and then gradually rose in December until the third lockdown at the end of December/beginning of January 2021.

The average changes in the first lockdown period were –84% for public transport, –76% for walking and –72% for driving. For the duration of the second lockdown, the corresponding changes were –64% for public transport, –47% for walking and –43% for driving. For the entire year, since 13 January 2020, the average changes were –51% for public transport, –33% for walking and –27% for driving.

#### 3.1.3 Edinburgh

The first lockdown in Edinburgh showed greater decrease in driving, public transport and walking than Greater London (Figure 2). The maximum measured change in public transport was –92%, in walking was –87% and in driving was –83%. Following the lockdown period, both driving and walking rose in harmony to their pre-lockdown (baseline) values, whereas public transport remained well below the pre-lockdown values until the end of the year. In this respect, the development of the mobility modes following the first lockdown is very different from that of Greater London, but similar to that observed for Cardiff and Belfast (Figure 2), for the reasons given above.

The formal second lockdown in Edinburgh was from 20 November to 11 December, while other mobility measures had been taken earlier. It follows that the formal lockdown period is less marked in the mobility curves for Edinburgh than for either Greater London or Cardiff, but similar to that for Belfast (Figure 2). During the second lockdown, the maximum measured change in public transport was –64%, in walking was –64% and in driving was –50%. In contrast to Greater London and Cardiff, there was no significant rise in mobility towards the end of the year: primarily because the formal lockdown was so late in the year.

The average changes in the first lockdown period were –89% for public transport, –82% for walking and –75% driving (Table 1). For the duration of the second lockdown, the corresponding changes were –59% for public transport, –58% for walking and –39% for driving. For the entire year from 13 January 2020, the average changes were –52% for public transport, –42% for walking and –29% for driving.

#### 3.1.4 Belfast

During the first lockdown in Belfast the maximum measured change in public transport was –88%, in walking was –79% and in driving was –76% (Figure 2). Following the lockdown period, both driving and walking rose in harmony to well above their pre-lockdown (baseline) values, while public transport remained well below the pre-lockdown values until the end of the year. These variations in transport are very similar to those for Cardiff and Edinburgh (Figure 2).

The formal second lockdown in Belfast was from 27 November to 11 December, but other mobility measures had been taken earlier. It follows that, as for Edinburgh, the formal lockdown period is less marked in the mobility curves than for Greater London and Cardiff (Figure 2). During the second lockdown, the maximum measured change in public transport was –67%, in walking was –63% and in driving was –40%. All these modes of transport stayed low until the end of the year, except for a rise in walking towards the end of December.

The average change in mobility during the first lockdown period was –83% for public transport, –68% for walking and –62% driving (Table 1). For the duration of the second lockdown, the corresponding changes were –61% for public transport, –42% for walking and –19% for driving. For the entire year from 13 January 2020, the average changes were –47% for public transport, –29% for walking and –16% for driving. On average, the reductions in walking and driving for the entire year are considerably less in Belfast than in the other cities.

### 3.2 Changes in Ground-Level NO_2_ Concentrations

**Figure 3:**
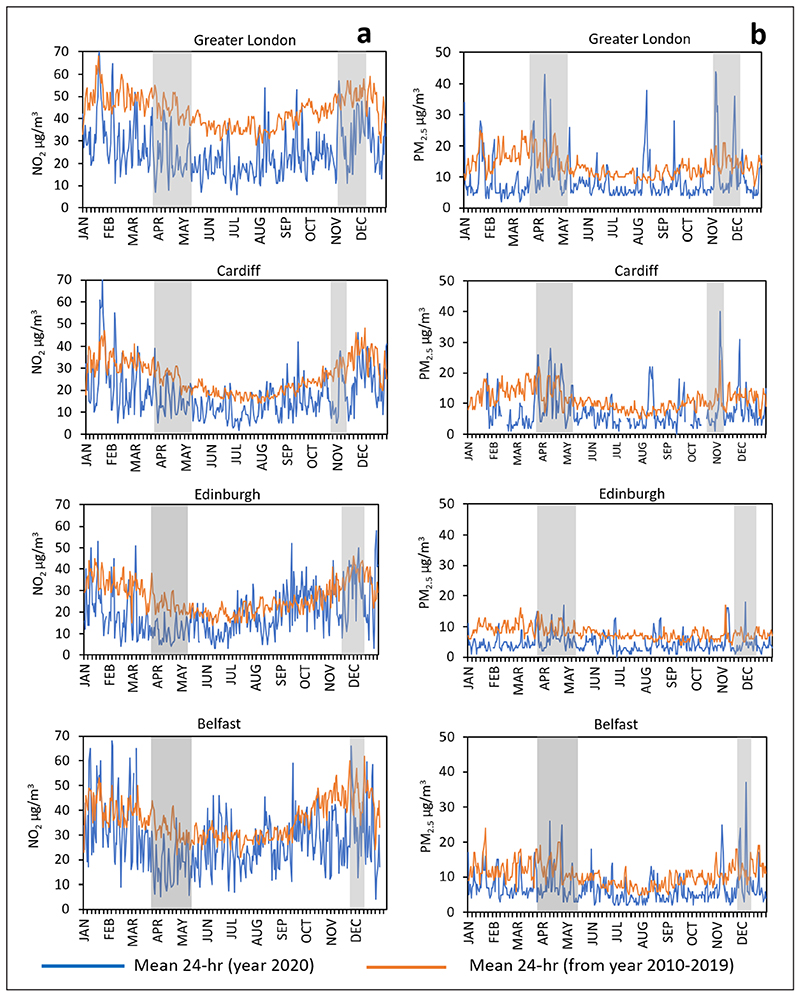
Mean daily time-series analysis of the air pollutants (a) NO_2_ and (b) PM_2.5_ for 2020 and the period 2010–19 (10 years as the baseline) for each of the four cities. *Note:* Dark grey highlighted columns show the periods of the first and second lockdowns.

**Figure 4:**
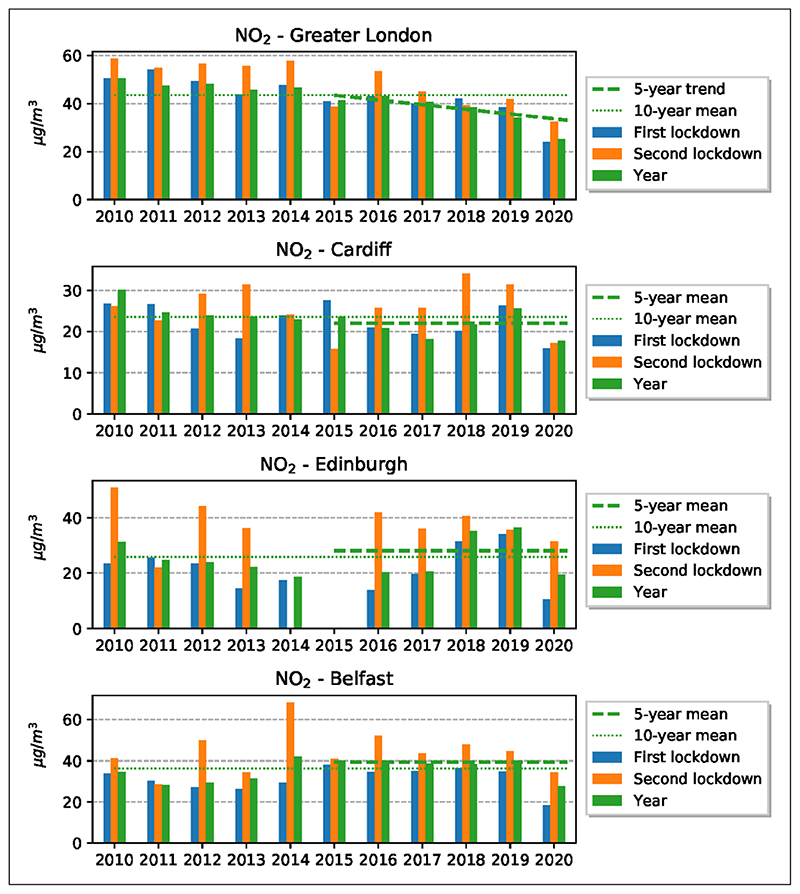
Comparison between the yearly average concentration of NO_2_ for all stations for each city during the two lockdown periods and the previous 10 years. *Note:* Dashed lines show the 10-year averages (2010–19); and broken lines show the five-year mean (trend for Greater London).

The results show that NO_2_ decreased significantly from 13 January until the end of December 2020 in comparison with the 10-year baseline (Figure 3a). On average, the 10-year baseline curve is above the 2020 curve for all four cities. The reduction with reference to the 10-year baseline, while clear, is quite irregular (blue line), presumably partly due to the effects of meteorological factors and different air masses—associated with different temperatures, moisture and wind speed (see Figures S1-S3 in Appendix A in the supplemental data online) at the location of the cities in 2020 (see Section 3.4). Such variability is reduced in the 10-year averages (brown line), which tend to smooth out pollution peaks and troughs. Also considered were the changes in NO_2_ concentrations in the two formal lockdown periods and the whole year for the four cities in comparison with the five-year trend (for Greater London), five-year mean (for the other cities) and mean 10-year concentrations in the same periods for all the stations (Figure 4) and for the urban-background stations (see Figure S5 in Appendix C in the supplemental data online). While the dates for the first lockdown were the same for all the cities, namely 23 March–10 May, the dates for the second lockdown were different.

The results show that for all the cities the reduction in NO_2_ was greater in the first than in the second lockdown (Figure 4). This is as expected because the first lockdown reduced the mobility much more than the second lockdown (Figure 2 and Table 1). Comparison with the same periods in the previous 10 years shows that the NO_2_ concentrations in the lockdown periods in 2020 were mostly lower than in the previous years. The only exceptions to this general conclusion are the second lockdown periods in Belfast and Edinburgh, where the NO_2_ concentrations were similar to, or slightly higher than, the two to three previous periods. This is understandable because driving was less reduced during the second lockdown in these two cities, particularly in Belfast, than in Greater London and Cardiff (Figure 2), and the exhaust from fossil fuel vehicles is one of the main contributors to NO_2_ concentration in urban areas.

The estimated relative changes in NO_2_ in the four cities for the two lockdown periods as well as for the whole year 2020, using the average 10-year concentrations in the same periods as the baseline, are presented in Table 2. In the first lockdown, the changes in NO_2_ concentrations for the four cities range from –36% to –54%, when all the monitoring stations are used. When only the urban-background stations are used (Table 2, in parentheses), then the change is from –35% to –53%. Thus, the results are very similar whether all the stations or only the urban-background stations are used. For the second lockdown, the change is from –18% to –46% or, for only the urban-background stations, from –21% to –53%, so rather similar in both cases. For the entire year, the change in NO_2_ concentration is from –23% to 42% (from –24% to –49%) for urban-background stations. Clearly, therefore, the lockdowns and the general mobility restrictions in 2020 resulted in significant overall reduction in the concentration of NO_2_.

The annual mean concentration of NO_2_ for the past 10 years in comparison with the mean for 2020 shows the impact of the general trend in concentrations since 2010. The annual mean concentrations for all four cities in 2020 are lower than those in any of the previous 10 years (Figure 4; and see Figure S5 in Appendix C in the supplemental data online). Furthermore, the results show clearly that the mean yearly concentration of NO_2_ in these four cities has been gradually decreasing in the past decade.

### 3.3 Changes in Ground-Level PM_2.5_ Concentrations

The results for PM_2.5_ are less clear than those for NO_2_, possibly because PM_2.5_ concentrations are more strongly affected by weather patterns and have more natural sources than NO_2_. While the average PM_2.5_ concentration in 2020 is generally lower than the average for the 10-year baseline period (Figure 3b), there are many concentration peaks throughout the year where, for a short while, the concentration is higher than the corresponding 10-year baseline. Such peaks may be partly related to sudden changes in wind direction and/or speed or other weather factors that bring different air masses from outside over large parts of the UK (Macintyre *et al*. 2016) (cf. Section 3.4).

**Figure 5:**
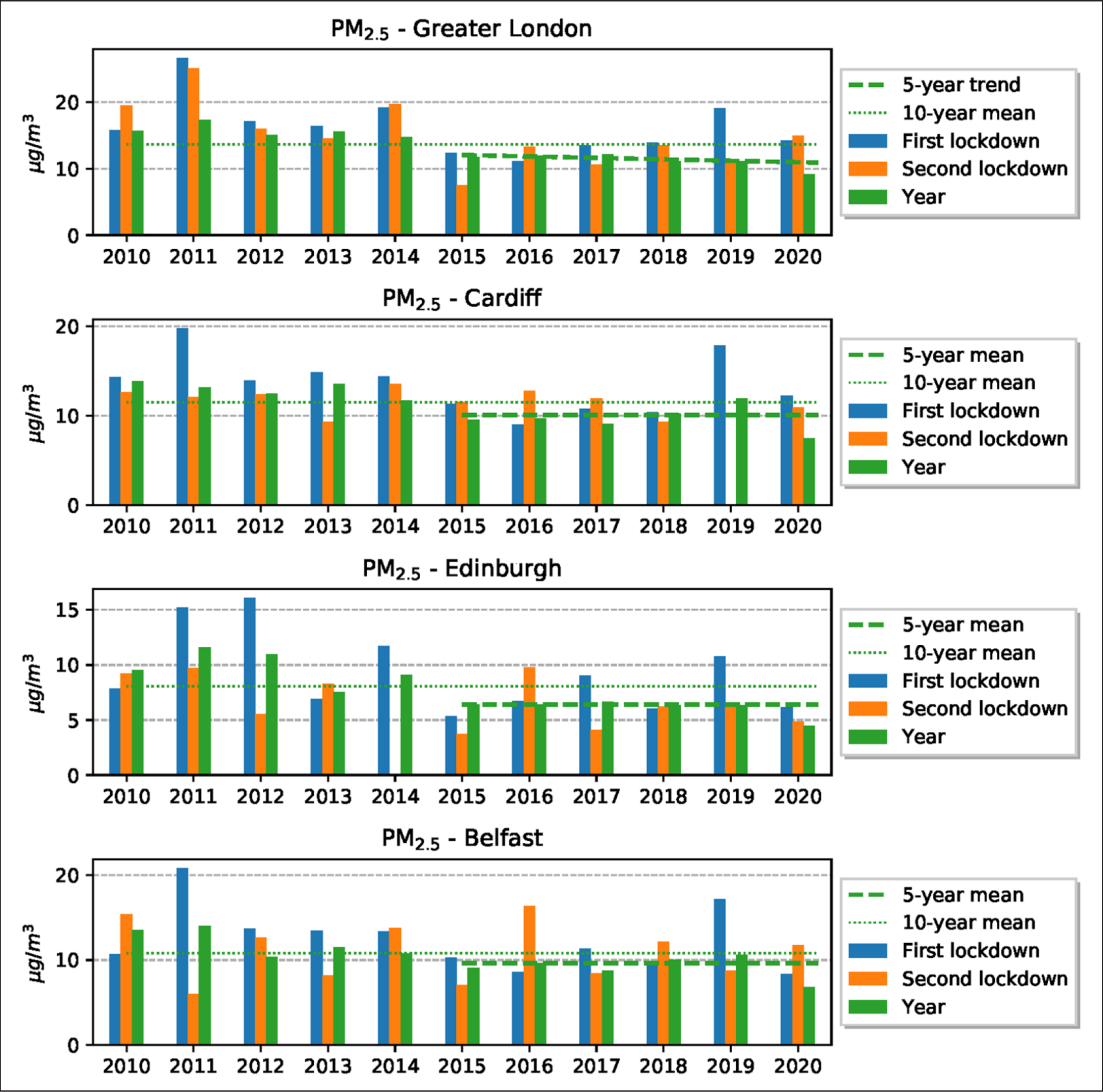
Comparison between the yearly average concentration of PM_2.5_ for all stations for each city and for the two lockdown periods. *Note:* Dashed lines show the 10-year average (2010–19); and broken lines show the five-year mean (trend for Greater London).

The changes in PM_2.5_ concentrations in the two lockdown periods were also compared with the mean 10-year concentrations in the same periods. The results (Figure 5; and see Figure S6 in Appendix C in the supplemental data online) show that for Greater London and Belfast the reduction in PM_2.5_ was much greater in the first lockdown than in the second, but for Cardiff and Edinburgh the reductions were slightly greater in the second lockdown. Comparison with the same periods in the previous 10 years shows that the PM_2.5_ concentration for the first lockdown in Belfast is the lowest, and for Greater London and Edinburgh the second to third lowest concentrations measured during these 10 years. However, the concentration in the second lockdown, while low in Edinburgh, is comparatively high in Cardiff and Belfast, and higher than in any of the same periods in the previous 10 years in Greater London. Thus, while overall the values for PM_2.5_ for the lockdown periods when considered together are low in comparison with the same periods in the previous decade, these comparatively low values are not uniform and the concentrations show great variations between the cities and between the lockdown periods.

The changes in PM_2.5_ in the four cities for the two lockdown periods as well as for the whole year 2020, using the average 10-year concentrations in the same periods as the baseline, are given in Table 2. In the first lockdown, the changes in PM_2.5_ concentrations for the four cities range from –5% to –74%, when all the monitoring stations are used. However, when only the urban-background stations are used (Table 2, in parentheses), then the change is from –4% to –37%. Thus, the results are different depending on whether all the stations or only the urban-background stations are used. For the second lockdown, the change is from –10% to –88% or, for only the urban-background stations, from –0% to –24%, so, again, different depending on the stations used. For the entire year (with urban-background stations in parentheses), the change in PM_2.5_ concentrations is from –31% to 82% (from –28% to –42%). Clearly, therefore, the lockdowns and the general mobility restrictions in 2020 resulted in an overall, even if highly variable, reduction in the concentration of PM_2.5_.

The yearly concentration of PM_2.5_ for the past 10 years in comparison with the mean for 2020 shows that the mean concentration for all four cities is lower in 2020 than in any of the previous 10 years. The data support the conclusion that the restriction to human mobility, and particularly the two lockdowns, in 2020 resulted in significant, even if somewhat variable, reduction in the atmospheric concentration of PM_2.5_ in the four cities considered in this study.

### 3.4 Weather-Corrected Air Pollutions Changes

In order to consider the effects of variable weather conditions, models using two machine learning (ML) algorithms—linear regression (LR) and gradient boosting regression (GBR)—were run to predict what air pollutant concentrations were expected to be during the Covid-19 period based on meteorological data and concentrations of air pollution from previous years. The daily absolute values (μg m^−3^) for NO_2_ and PM_2.5_ are predicted based on the LR and GBR models. The average yearly absolute values for the two models are given in (Table 3).

The relative changes or anomalies for 2020, that is, the differences between the predicted and observed values for NO_2_ and PM_2.5_, are given in Table 3. As indicated, the anomalies are calculated through two different ML algorithms, LR and GBR, the latter being regarded as more reliable because of somewhat lower mean absolute errors—a metric for the model’s performance (Table 3). For LR, all anomalies and, for GBR, all except one, are negative, indicating that the measured concentrations of NO_2_ and PM_2.5_ are lower than would be expected from the weather conditions at the time. For GBR, the NO_2_ anomalies are –21% for Greater London, –19% for Cardiff, –41% for Edinburgh and –27% for Belfast. Similarly, the PM_2.5_ anomalies are +7% for Greater London, –1% for Cardiff, –15% for Edinburgh and –14% for Belfast.

**Figure 6:**
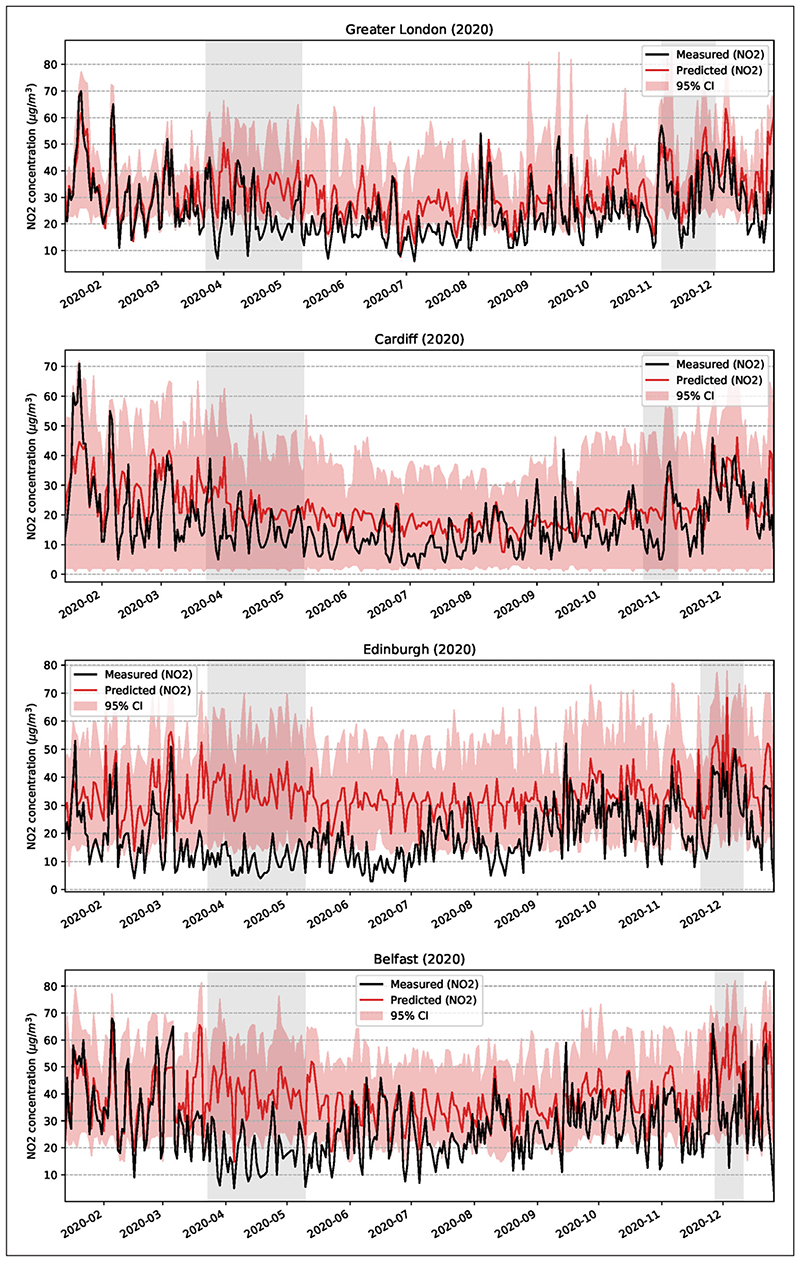
The 95% confidence intervals for the weather-corrected machine learning (ML) model for the four cities and for the predicted NO_2_.

More specifically, the estimated 95% confidence intervals for NO_2_ and PM_2.5_ for all four cities indicate that the (mostly) reduced levels of pollution cannot be explained in terms of weather conditions. They are very likely attributed to other factors, primarily the reduction in mobility. The confidence intervals for the predicted NO_2_ and for the four cities are shown in Figure 6.

### 3.5 Correlation Between Mobility and Weather-Corrected Pollution Changes

**Figure 7:**
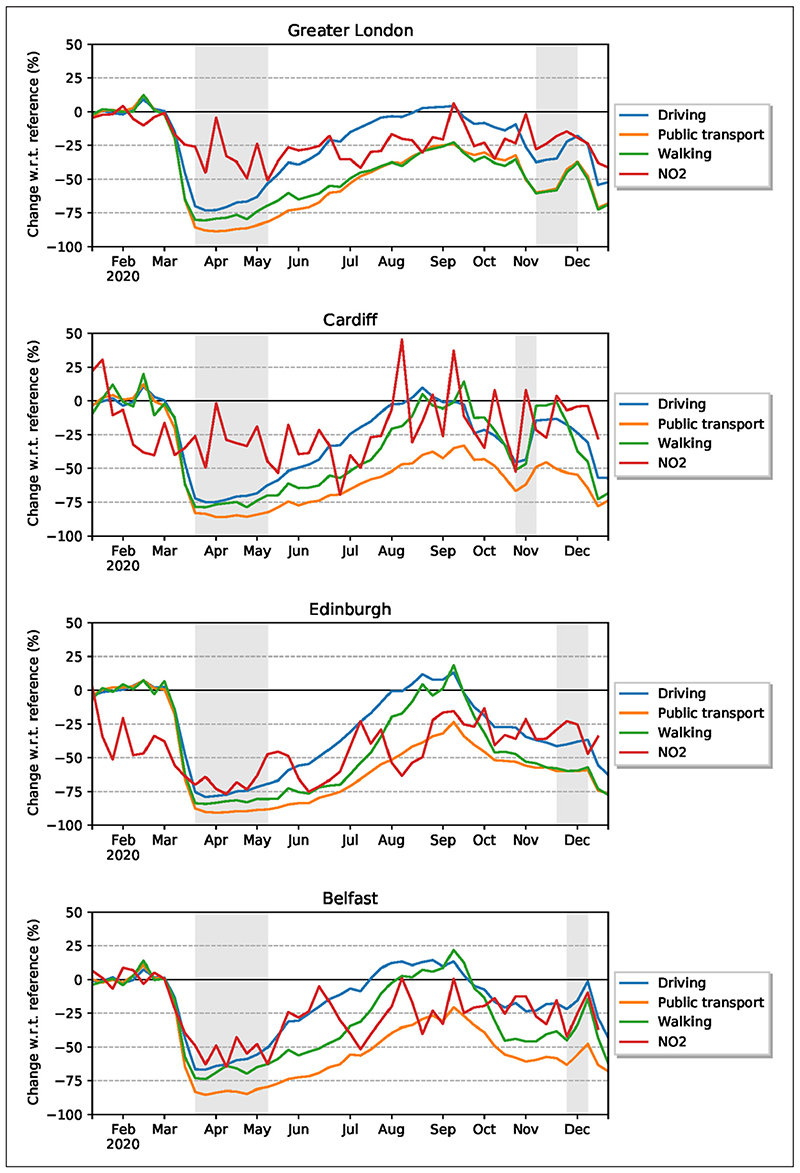
Air pollution anomalies (relative change in percentage compared with the baseline) for NO_2_ (weather-corrected models) for each city in relation to changes (%) in driving, public transport and walking. *Note:* For improved clarity, both mobility data and air pollution data are aggregated weekly to smooth out the curves.

While the results indicate that concentrations of both NO_2_ and PM_2.5_ in the four cities in 2020 were reduced as a result of the restrictions on human mobility, further analyses are warranted as to the correlation between the changes in mobility and the changes in the concentrations of the pollutants. It turns out that the correlations between mobility and the five-year mean daily and weekly ground-level concentrations are not high, although significant. Analysis of the correlations between changes in weekly concentration (using weather-corrected models) and weekly mobility activities indicate a strong correlation between mobility activities and the predicted concentrations for NO_2_. In particular, there is a significant correlation between predicted NO_2_ reduction and vehicle driving (generally *p* < 0.05) and public transport (generally *p* < 0.01) for all the cities in both models (Figure 7 and Table 4).

The strongest correlation is between the reduction in NO_2_ concentrations and mobility for Belfast with correlation coefficients *r_s_* = 0.73 for public transport (*p* < 0.0001) and *r_s_* = 0.64 for driving (*p* < 0.0001). The correlations between NO_2_ and public transport significantly increase from the five-year measurement data to the modelled (predicted) data from *r_s_* = 0.18 to 0.69 for Greater London, from *r_s_* = 0.24 to 0.57 for Edinburgh and from *r_s_* = 0.59 to 0.73 for Belfast (public transport), all values being statistically significant (Table 4). For Cardiff correlations increase by a smaller margin (from *r_s_* = 0.33 to 0.34, *p* < 0.05 for public transport), presumably because there only temperature data were available to train the models.

There are, however, no significant correlations between PM_2.5_ and public transport and driving (Table 4). This suggests that PM_2.5_ concentrations were less affected by transportation changes during the lockdowns than the NO_2_ concentrations. There are also some negative relationships between PM_2.5_ and mobility. The negative correlations are somehow counter-intuitive because they suggest that PM_2.5_ increases as mobility decreases. The results show that while there is a significant and strong correlation between NO_2_ and mobility (using weather-corrected models), no such correlation exists for PM_2.5_, presumably primarily because sources of pollution from PM_2.5_ are more strongly related to industry, power generation and residential energy use than to traffic. For example, much of PM measured in Greater London originates outside the city (Saunders *et al*. 2012). Thus, the constant movement of air masses and the different types of sources for PM_2.5_, many of which are outside the four cities where the concentrations were measured, make for a less clear correlation with the mobility changes in the cities in 2020.

## 4 Impacts on Health and Mortality

Air pollution has a negative impact on health and is considered a major contributor to premature mortality. In the UK somewhere between 28,000 and 36,000 premature deaths every year are attributable to poor air quality (PHE 2019). The attributable fraction (AF) of all-cause mortality associated with NO_2_ and PM_2.5_ for each year 2010–20 is estimated below to assess the potential health impacts of the lockdown-induced reductions in air pollution in 2020.

At the local level, the UK Committee on the Medical Effects of Air Pollutants (COMEAP) (2015), recommends three metrics for calculating the mortality burden associated with particulate air pollutants. One of these is ‘attributable fraction’ (AF), which is the proportion of local deaths attributable to the long-term (more than one year) exposure to anthropogenic air pollution. The AF of all-cause mortality in each city associated with PM_2.5_ and NO_2_ for 2020 and the each of the 10 years before is calculated as follows (COMEAP 2015; Saunders *et al*. 2012; Macintyre *et al*. 2016):

(3) AF=(RR−1)/RR

where RR is the relative risk. Attributable fraction is often expressed as a percentage and, therefore, calculated as:

(4) AF=100×(RR−1)/RR

and is equal to:

(5) $$\text{RR}=\beta^{\left(x/10\right)}$$

where *β* is the concentration-response coefficient for each pollutant (per 10 μg m^−3^); and *x* is the annual average pollutant concentration. Not provided is an estimate of changes in absolute mortality figures from air pollution due to lack of daily baseline all-cause mortality data. It is worth noting that the direct impact of Covid-19 would result in elevated baseline mortality figures. The *β*-values for estimating the annual attributable mortality from long-term exposure to air pollution used here are based on COMEAP’s recommendations. Specifically, the *β*-coefficient for PM_2.5_ used here is 1.060 (95% confidence interval (CI) = 1.04–1.08), based on COMEAP (2010), and for NO_2_ is 1.023 (95% CI = 1.008–1.037) from COMEAP (2018).

Using equations (3–5) and the information above, the AFs (%) were calculated based on the yearly average NO_2_ and PM_2.5_ since 2010 and then compared with the calculated AF for 2020 with those of the previous decade. The results show as follows (Table 5). First, for all four cities, there has been a gradual decline in the NO_2_-related AFs since 2010. The decline is not strictly linear—there have been some occasional increases from year to year, such as in both London and Belfast—but overall the AF related to this pollutant has decreased. In 2010, the AF due to NO_2_ is estimated to be 7–10% for these four cities, but falls to 5–7% in 2019. A further decline then occurred in 2020, reducing the NO_2_-related AF to 3–5%.

Second, for the PM_2.5_-related AFs, the general results are similar to those for NO_2_: there has been an overall decline in the AFs since 2010 (Table 5). Again, the decline is not linear, with many slight increases from year to year in all four cities. In particular, the PM_2.5_-related changes in AF in both Cardiff and Belfast are irregular, while Greater London and Edinburgh show a steadier decline in AF. In 2010, the PM_2.5_-related PMs are estimated at 6–8% for the four cities, but decline to 3–6% in 2019. Again, a further decline occurs in 2020 when the PM_2.5_-related PM is 2–5%.

## 5 Discussion

Covid-19-related mobility restrictions in four cities in the UK were compared with the associated changes in the concentrations of NO_2_ and PM_2.5_. The three measured mobility factors—driving, public transport and walking—show a sharp decrease during and following the first lockdown in all the cities. The greatest change was in public transport, where the maximum measured change was –92% (Edinburgh), followed closely by Greater London (–90%), while the least change was in driving. More specifically, the maximum change in driving was from –76% (Belfast) to –83% (Edinburgh). Following the lockdowns there was a gradual increase in these modes of transport, and both walking and driving reached close to or above their pre-lockdown values—mostly in September–October—before the second lockdown.

Studies in other countries where Covid-19-related mobility restrictions were imposed show similar changes in mobility. For example, Ash’aari *et al*. (2020) report a sharp decrease in visits to parks, transit stations and workplaces during lockdowns in Malaysia in early 2020. Also, Li & Tartarani (2020) report a great reduction in visits to transit stations, workplaces, car parks and driving during lockdown in Singapore in the early part of 2020.

The second lockdown was at different times in the four cities, and its effects on human mobility were neither as abrupt nor as great as in the first lockdown. The greatest change, again, was in public transport, where the maximum change was from –63% (Greater London) to –76% (Cardiff). Similarly, the maximum change in driving was from –40% (Belfast) to –60% (Cardiff).

The average reductions in NO_2_ concentrations in 2020 are significant in comparison with average concentrations in the cities in the previous 10 years (Figure 3a). Also, the NO_2_ concentrations in the specific lockdown periods in 2020 show much reduction in comparison with the same periods in the previous 10 years. While there has been a gradual decrease in NO_2_ in the atmosphere in the UK during the past decade, the reduction in 2020 is greater that would be expected if that trend had simply continued at the same rate as before (Figures 4 and 5). For urban-background monitoring stations, the changes in NO_2_ in the four cities range from –35% to –53% in the first lockdown, and from –21% to –53% in the second lockdown. For the entire year the changes in NO_2_ at urban-background stations range from –24% to –49%.

While PM_2.5_ also shows an overall reduction during 2020, the results are not as clear as for NO_2_. The average PM_2.5_ changes in the first lockdown in the four cities at the urban-background stations range from –4% to –37%. For the second lockdown, the changes, again using the urban-background stations, are from 0% to –24%. For the entire year 2020 for urban-background stations, the changes in PM_2.5_ concentration are from –28% to –42%. The results also show that the yearly concentrations of PM_2.5_ in the past decade are higher in these cities than in 2020. Thus, the restrictions to human mobility in 2020 in these four cities resulted in significant, even if somewhat variable, reduction in the atmospheric concentration of PM_2.5_.

Several studies have considered the effects of various Covid-19-related mobility restrictions on the concentrations of various pollutants in the atmosphere in various countries and cities. For example, Baldasano (2020) considered the effects of lockdowns in early 2020 on the concentration of NO_2_ in Barcelona and Madrid in Spain and concluded that the changes in NO_2_ concentrations were –50% and –62%, respectively. Similarly, Li & Tartarani (2020) considered the early 2020 lockdown effects on various pollutants in Singapore. As for NO_2_, the changes were –54%, and for PM_2.5_, the changes were –29%. Also, Ash’aari *et al*. (2020) analysed the changes in several pollutants during similar mobility restrictions in Malaysia in early 2020. They found that the NO_2_ concentrations changed by –54% and PM_2.5_ concentrations by –23.1%. Thus, the results for Malaysia are very similar to those for the adjacent Singapore. For the world as a whole, Venter *et al*. (2020) used more than 10,000 air quality stations in 34 countries to measure the effects of various lockdowns in early 2020 on the concentrations of NO_2_ and PM_2.5_. They found that, on average, the change in NO_2_ during the lockdowns was –60% and that of PM_2.5_ was –31%.

Using the weather-correction ML technique, all four cities show NO_2_ and PM_2.5_ concentration anomalies in 2020. These anomalies are the difference between the measured and predicted concentrations. The anomalies were calculated through two different ML algorithms, LR and GBR, the latter being regarded as more reliable because of somewhat lower mean absolute errors. For LR, all anomalies, and for GBR, all except one, are negative, so that the measured concentrations of NO_2_ and PM_2.5_ are lower than would be expected from the weather conditions. For GBR, the NO_2_ anomalies are –21% for Greater London, –19% for Cardiff, –41% for Edinburgh and –27% for Belfast, while the PM_2.5_ anomalies are +7% for Greater London, –1% for Cardiff, –15% for Edinburgh and –14% for Belfast. All the negative anomalies are presumably the results of the Covid-19 mobile restrictions.

The effects of excess concentrations of PM_2.5_ and NO_2_ in the atmosphere on human health are well documented (Saunders *et al*. 2012; Carnell *et al*. 2019; Quarmby *et al*. 2019). In particular, many premature deaths are attributed to these pollutants (EEA 2020). Therefore, the effects of the changes in NO_2_ and PM_2.5_ concentrations in the four cities in 2020 on the percentage of attributable mortality (AF) due to exposure to these pollutants were calculated. The results were compared with those for the previous decade and the general trend in the concentration of these pollutants during that decade. The results (Table 5) show a significant decline in AF in 2020. This decline is more than would be expected from the overall decrease in AF due to improved air quality in the past decade. This decline was also tested, and confirmed, by weather-corrected models. Therefore, it can be concluded that the restrictions in human mobility in 2020 and the associated decrease in concentration of NO_2_ and PM_2.5_ resulted in a significant reduction in associated AF and had, by implication, other positive effects on public health.

## 6 Conclusions

The present study indicates significant positive short-term effects of city-scale Covid-19-related reductions in transportation on ground-level concentrations of nitrogen dioxide (NO_2_) and particulate matter PM_2.5_. While there is a significant correlation between the decline in NO_2_ concentrations and public transport (*p* < 0.05) and vehicle driving (*p* < 0.05), no significant correlation is found for changes in PM_2.5_ concentrations (in weather-corrected models). This suggests that NO_2_ concentrations are more strongly affected by changes in the volume of on-land transportation than PM_2.5_ concentrations. For these cities, the concentration of PM_2.5_ is likely to depend partly on residential energy use, power generation and agriculture, and partly on the general complexity of its formation.

Finding the means to curb air pollution remains important. Empirical evidence at a city scale reveals correlations between reduction in on-land transport and changes in the ambient PM_2.5_ and NO_2_ concentrations. The results provide justification for city-level initiatives to reduce vehicular traffic. Well-designed and effective policy interventions can substantially reduce long-term air pollution and have positive health impacts.

## Supplementary Material

Supplementary material Supplemental data for this article can be accessed at: DOI: https://doi.org/10.5334/bc.124.s1

## Figures and Tables

**Table 1:** Average mobility reduction (for driving, public transport and walking) for the entire year 2020 and the two lockdown periods for the four cities.

City	Period	Dates	Average Mobility Reduction (%)		
			Driving	Public Transport	Walking
Greater London	Year	13 January–30 December	–23%	–45%	–43%
	First lockdown	23 March–10 May	–69%	–86%	–78%
	Second lockdown	5 November–2 December	–36%	–58%	–59%
Cardiff	Year	13 January–30 December	–27%	–51%	–33%
	First lockdown	23 March–10 May	–72%	–84%	–76%
	Second lockdown	23 October–9 November	–43%	–64%	–47%
Edinburgh	Year	13 January–30 December	–29%	–52%	–42%
	First lockdown	23 March–10 May	–75%	–89%	–82%
	Second lockdown	20 November–11 December	–39%	–59%	–58%
Belfast	Year	13 January–30 December	–16%	–47%	–29%
	First lockdown	23 March–10 May	–62%	–83%	–68%
	Second lockdown	27 November–11 December	–19%	–61%	–42%

*Note:* Apple mobility data are normalised by taking 62 days from 13 January to 14 March as a baseline. *Source:* Apple Mobility Reports.

**Table 2:** Average air-pollution reduction (NO_2_ and PM_2.5_) for all ground-level monitoring stations as well as urban-background stations separately (in parentheses) for the entire year 2020 as well as for two lockdown periods for the four cities.

City	Period	Dates	Air Pollution Reduction (%)	
			NO_2_ (Urban Background)	PM_2.5_ (Urban Background)
Greater London	Year	13 January–30 December	–42% (–38%)	–31% (–28%)
	First lockdown	23 March–10 May	–47% (–39%)	–13% (–8%)
	Second lockdown	5 November–2 December	–35% (–29%)	0% (0%)
Cardiff	Year	13 January–30 December	–32% (–41%)	–35% (–30%)
	First lockdown	23 March–10 May	–36% (–50%)	–5% (–4%)
	Second lockdown	23 October–9 November	–43% (–53%)	–10% (–10%)
Edinburgh	Year	13 January–30 December	–27% (–49%)	–42% (–42%)
	First lockdown	23 March–10 May	–54% (–53%)	–35% (–37%)
	Second lockdown	20 November–11 December	–18% (–38%)	–24% (–24%)
Belfast	Year	13 January–30 December	–23% (–24%)	–33% (–33%)
	First lockdown	23 March–10 May	–43% (–35%)	–33% (–32%)
	Second lockdown	27 November–11 December	–23% (–21%)	–14% (–14%)

**Table 3:** Two modelled predictions—linear regression (LR) and gradient boosting regression (GBR)—for air pollutants in 2020 compared with ground-level concentrations.

Annual Mean Concentration	2020 Ground-Level Measured Data (μg m^−3^)		LR Prediction (μg m^−3^)	Mean Absolute Error, LR (μg m^−3^)	Relative Change (%), LR Prediction	GBR Prediction (μg m^−3^)	Mean Absolute Error, GBR (μg m^−3^)	Relative Change (%), GBR, Prediction
No_2_	Greater London	25.3	30.6	5.88	–18%	31.9	5.31	–21%
	Cardiff	17.8	24.1	8.95	–26%	21.9	8.75	–19%
	Edinburgh	19.4	42.9	9.63	–55%	33.2	7.96	–41%
	Belfast	27.7	38.9	8.05	–29%	37.8	7.93	–27%
PM_2.5_	Greater London	9.2	9.7	4.36	–5%	8.6	3.73	+7%
	Cardiff	7.5	9.7	4.64	–23%	7.5	4.34	–1%
	Edinburgh	4.5	6.0	2.96	–26%	5.3	2.69	–15%
	Belfast	6.8	7.7	3.02	–12%	8.0	3.02	–14%

*Note:* Mean absolute errors and relative changes (%) are given for each model prediction.

**Table 4:** Spearman’s rank correlation coefficients (*r*_s_) between two predicted models for air pollutants (NO_2_ and PM_2.5_) and mobility (driving and public transport) for each of the four cities. The results are compared with the five-year mean daily and five-year mean weekly trends.

City	Pollutant	Spearman	Five-Year Mean (Weekly)		LR (Daily, Aggregated Weekly)		GBR (Daily, Aggregated Weekly)	
			DRIVING	PUBLIC TRANSPORT	DRIVING	PUBLIC TRANSPORT	DRIVING	PUBLIC TRANSPORT
Greater London	No_2_	^*r*^s	0.16	0.18	0.58	0.67	0.60	0.69
		*p*-value	0.26	0.22	<0.0001	<0.0001	<0.0001	<0.0001
	PM_2.5_	*r_s_*	–0.19	–0.30	–0.04	–0.10	–0.22	–0.26
		*p*-value	0.17	0.03	0.80	0.49	0.13	0.06
Cardiff	No_2_	^*r*^s	0.31	0.33	0.34	0.40	0.32	0.34
		*p*-value	0.02	0.02	0.01	<0.01	0.02	0.01
	PM_2.5_	*r_s_*	–0.10	–0.16	–0.28	–0.31	–0.22	–0.25
		*p*-value	0.48	0.26	0.05	0.03	0.13	0.09
Edinburgh	No_2_	*r_s_*	0.29	0.24	0.50	0.53	0.54	0.57
		*p*-value	0.04	0.10	<0.001	<0.0001	<0.0001	<0.0001
	PM_2.5_	^*r*^s	–0.09	–0.13	–0.11	–0.01	–0.22	–0.15
		*p*-value	0.52	0.37	0.47	0.93	0.14	0.33
Belfast	No_2_	*r_s_*	0.54	0.59	0.60	0.66	0.64	0.73
		*p*-value	<0.0001	<0.0001	<0.0001	<0.0001	<0.0001	<0.0001
	PM_2.5_	^*r*^s	0.09	–0.02	–0.28	–0.25	–0.27	–0.21
		*p*-value	0.55	0.91	0.07	0.10	0.07	0.16

*Note:* GBR = gradient boosting regression; and LG = linear regression.

**Table 5:** Health impact assessments for the four cites based on attributable fraction (AF) of all-cause mortality associated with NO_2_ and PM_2.5_ (%) for 2020 and the previous 10 years.

Pollutant	City	Af Of All-Cause Mortality Associated With Each Pollutant (%)										
		2010	2011	2012	2013	2014	2015	2016	2017	2018	2019	2020
NO_2_	Greater London	9.93%	8.90%	9.73%	8.49%	8.90%	7.86%	8.07%	8.07%	7.23%	6.81%	5.10%
	Cardiff	7.23%	5.95%	5.95%	5.74%	5.53%	5.95%	5.10%	4.45%	4.01%	4.88%	3.35%
	Edinburgh	6.81%	5.53%	5.31%	4.88%	4.23%	–	4.45%	4.45%	4.01%	4.66%	2.91%
	Belfast	7.65%	6.17%	6.38%	6.81%	6.17%	6.38%	6.59%	5.53%	5.95%	5.31%	4.01%
PM_2.5_	Greater London	8.37%	9.43%	8.90%	7.83%	8.37%	6.21%	6.21%	6.21%	6.21%	6.21%	5.11%
	Cardiff	7.83%	7.30%	6.75%	7.83%	6.75%	5.66%	5.66%	5.11%	5.66%	6.75%	4.00%
	Edinburgh	5.66%	6.75%	6.21%	4.55%	5.11%	3.44%	3.44%	4.00%	3.44%	3.44%	2.30%
	Belfast	7.30%	7.83%	5.66%	6.75%	6.21%	5.11%	5.66%	5.11%	5.66%	6.21%	4.00%
