# Supplementary material for: Covid-19 mobility restrictions: impacts on urban air quality and health
Source: Build Cities. Author manuscript; Available in PMC 2021 Oct 25. (PMC7611887; doi:10.5334/bc.124)
Supplement: Supplementary material [file EMS137022-supplement-Supplementary_Material.pdf]

Mohajeri, N., Walch, W., Gudmundsson, A., Heaviside, C., Askari. S., Wilkinson, P. & Davies, M. (2021) Covid-19 mobility restrictions: impacts on urban air quality and health. *Buildings & Cities.*

Supplemental data

Appendix A
Historical and year 2020 weather data

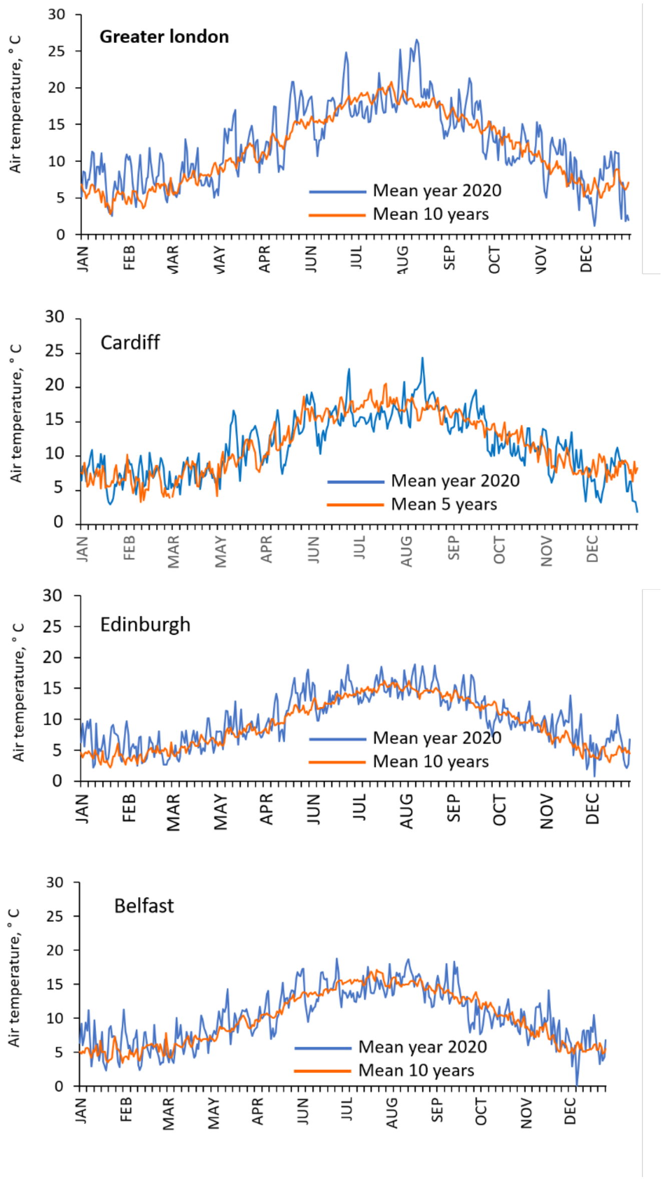

Figure S1. Mean daily temperature (°C) for the year 2020 and the previous 10 years (2010-2019) for 4 cities in the study

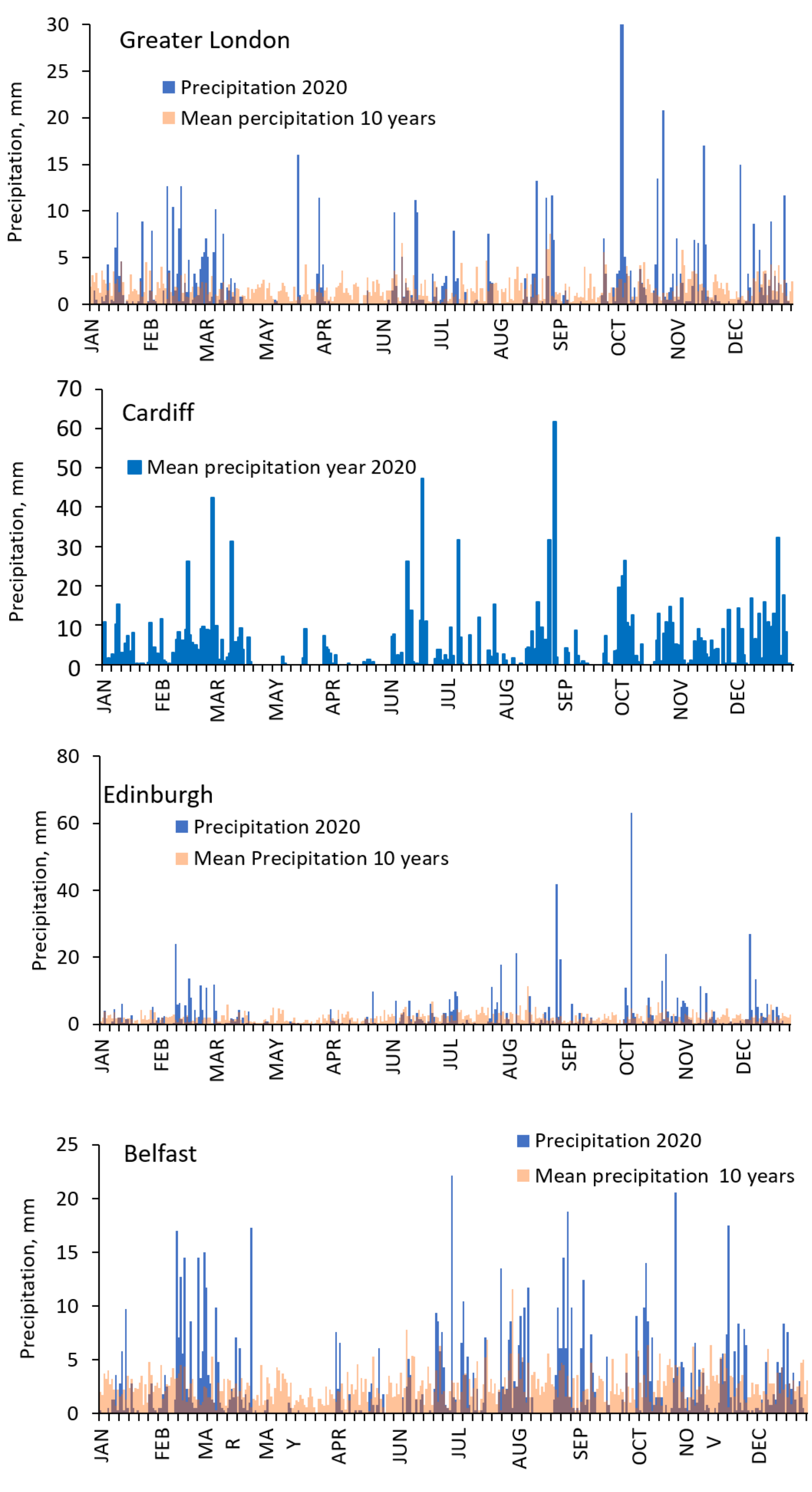

Figure S2. Mean daily precipitation (mm) for the year 2020 and the previous 10 years (2010-2019) for 4 cities in the study

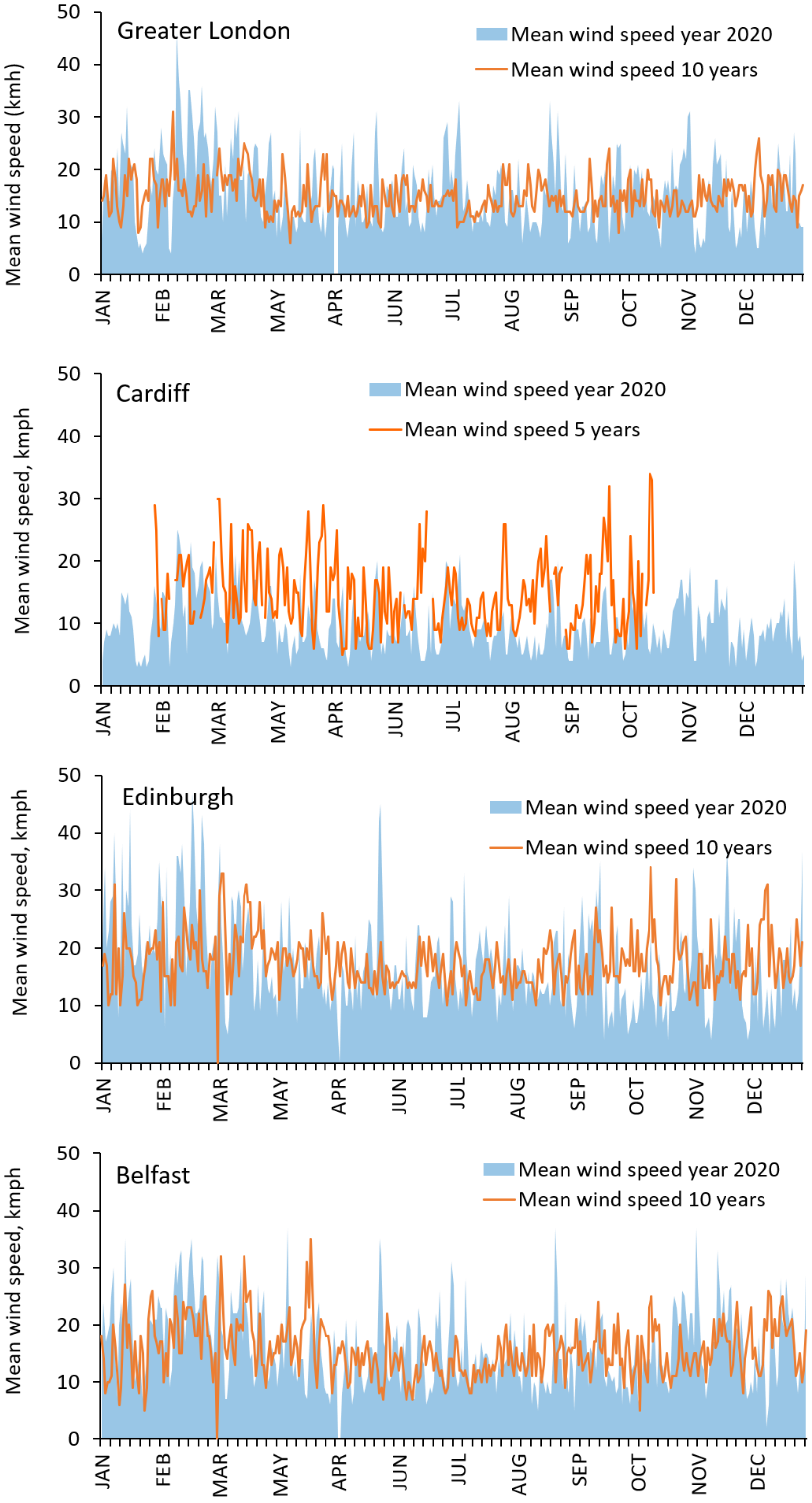

Figure S3. Mean daily wind speed (in kmph) for the year 2020 and the previous 10 years (2010-2019) for 4 cities in the study

Appendix B
Feature importance for weather-corrected Machine Learning model

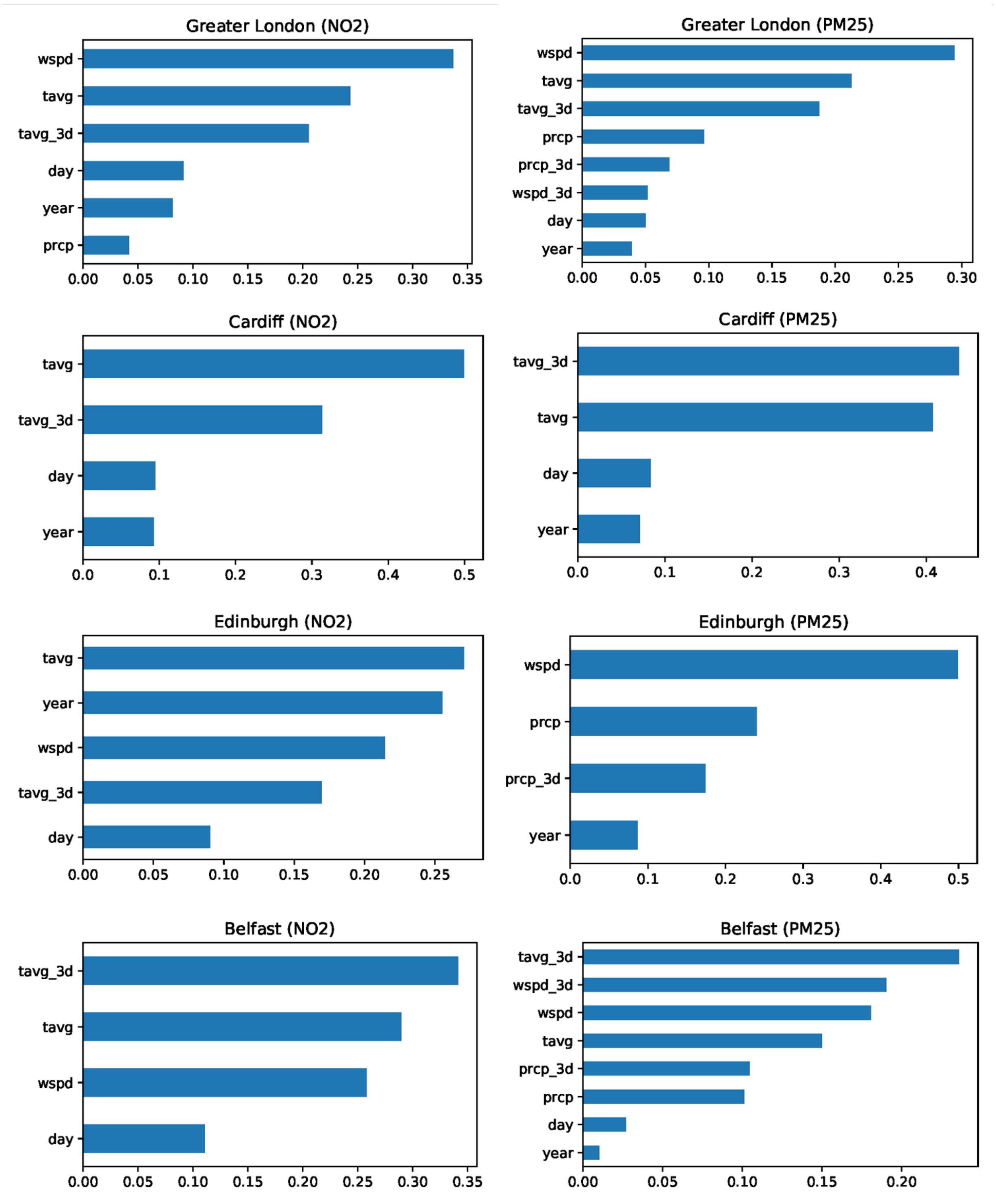

Figure S4. Feature importance for weather corrected Machine Leaning models for each city and for NO_2 and $PM_{2.5}$

Appendix C

Changes in the ground-level NO_2 and $PM_{2.5}$ concentrations (for urban background stations)

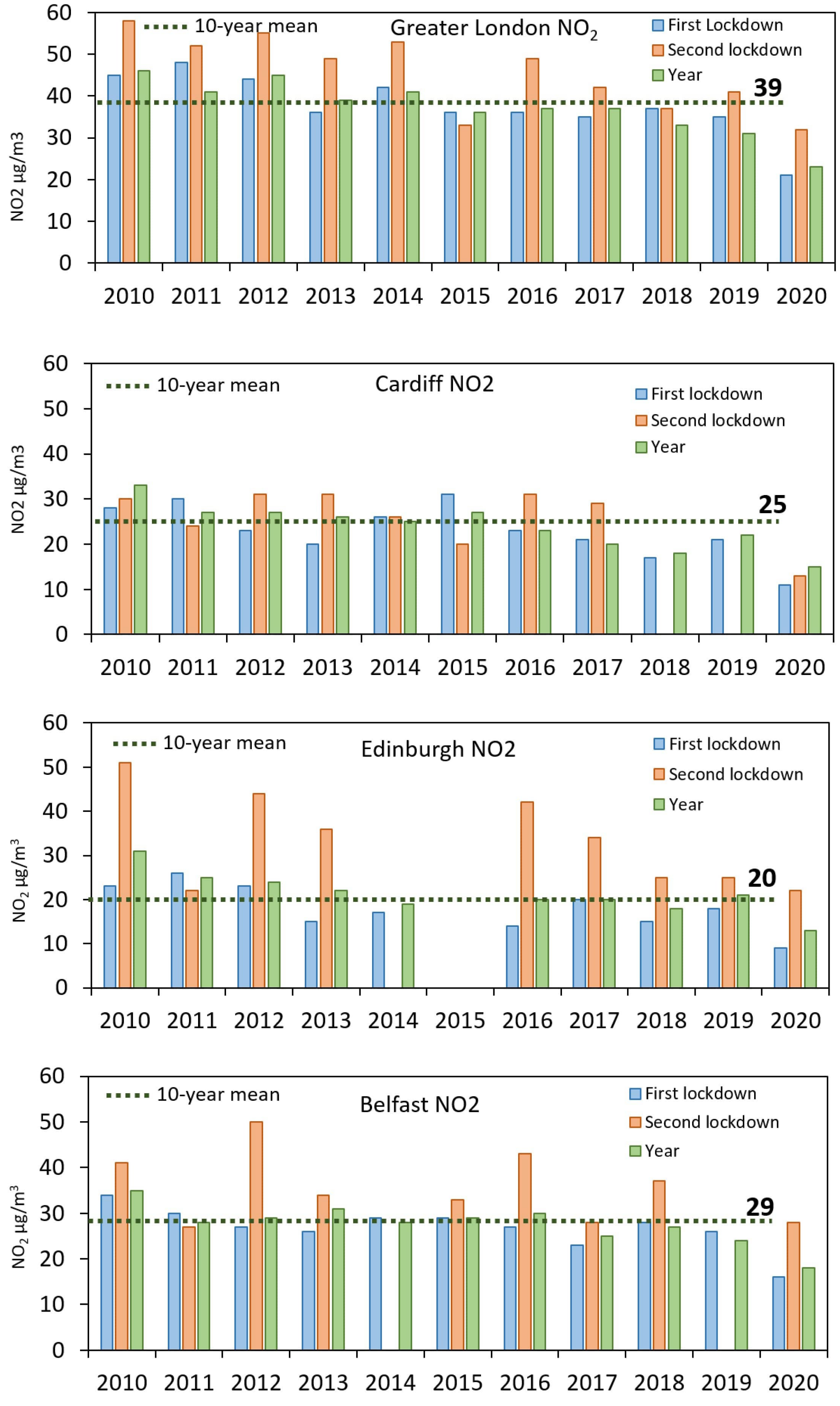

Figure S5. Comparison between yearly average concentration of NO_2 for urban background stations for each city and for the two lockdown periods. Dashed lines show the 10-year average (2010-2019).

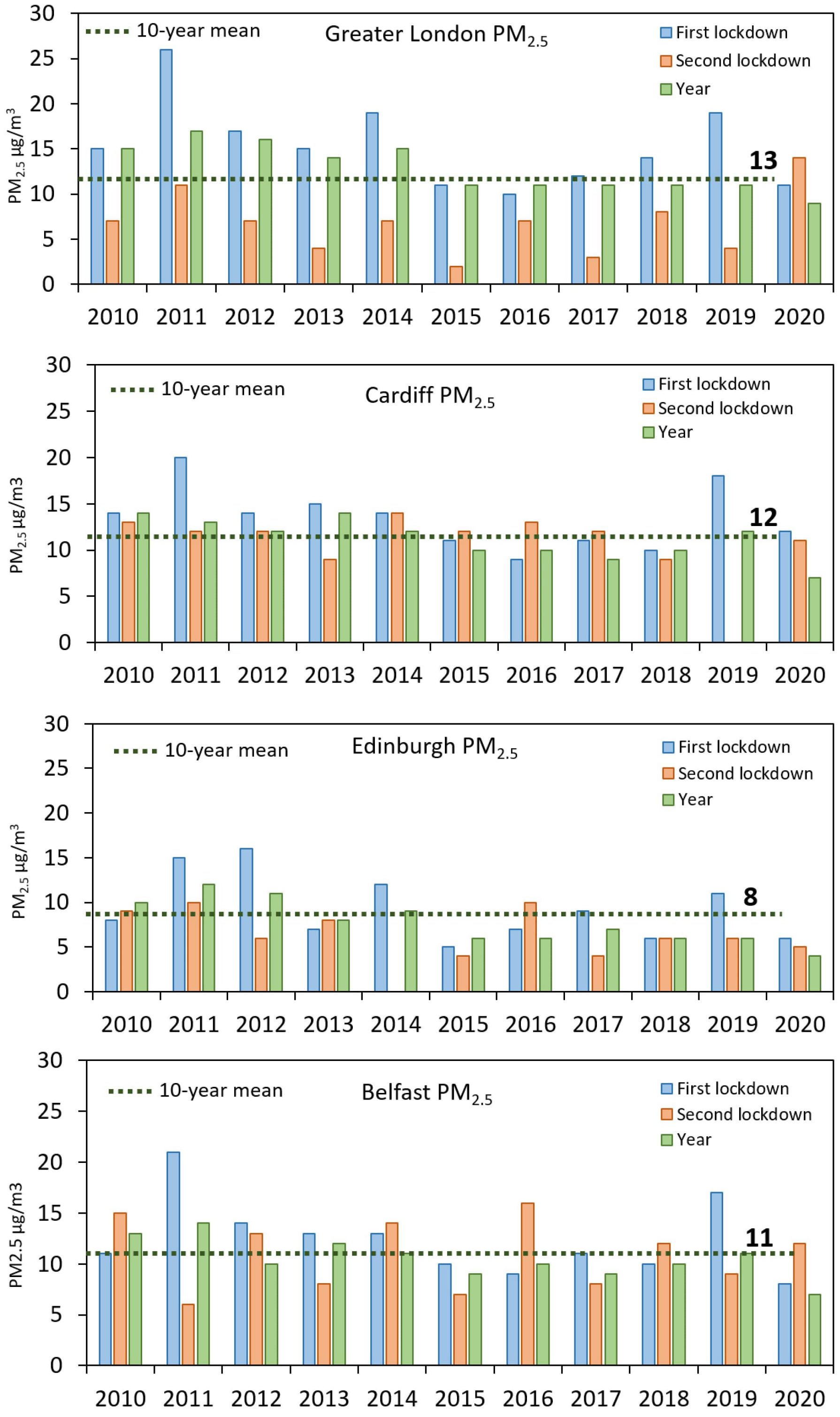

Figure S6. Comparison between yearly average concentration of $PM_{2.5}$ for urban background stations for each city and for the two lockdown periods. Dashed lines show the 10-year average (2010-2019).
